# The mitotic checkpoint is a targetable vulnerability of carboplatin-resistant triple negative breast cancers

**DOI:** 10.1038/s41598-021-82780-6

**Published:** 2021-02-04

**Authors:** Stijn Moens, Peihua Zhao, Maria Francesca Baietti, Oliviero Marinelli, Delphi Van Haver, Francis Impens, Giuseppe Floris, Elisabetta Marangoni, Patrick Neven, Daniela Annibali, Anna A. Sablina, Frédéric Amant

**Affiliations:** 1 VIB-KU Leuven Center for Cancer Biology, VIB, Leuven, Belgium; 2 Department of Oncology, KU Leuven and Leuven Cancer Institute (LKI), 3000 Leuven, Belgium; 3 School of Pharmacy, University of Camerino, Camerino, Italy; 4 VIB Center for Medical Biotechnology, Ghent, Belgium; 5 Department of Biomolecular Medicine, Ghent University, Ghent, Belgium; 6 VIB Proteomics Core, Ghent, Belgium; 7 Department of Imaging and Pathology, KU Leuven, Leuven, Belgium; 8 Department of Pathology, University Hospitals Leuven, KU Leuven, Leuven, Belgium; 9 Translational Research Department, Institut Curie, PSL Research University, Paris, France; 10 Department of Obstetrics and Gynecology, University Hospitals Leuven, 3000 Leuven, Belgium; 11 Division of Oncogenomics, The Netherlands Cancer Institute, Amsterdam, The Netherlands; 12 Centre for Gynecologic Oncology Amsterdam (CGOA), Antoni Van Leeuwenhoek-Netherlands Cancer Institute (AvL-NKI), University Medical Center (UMC), Amsterdam, The Netherlands

**Keywords:** Combination drug therapy, Breast cancer, Cancer therapeutic resistance, Chemotherapy, Breast cancer, Cancer models, Cancer therapy, Molecular medicine

## Abstract

Triple-negative breast cancer (TNBC) is the most aggressive breast cancer subtype, lacking effective therapy. Many TNBCs show remarkable response to carboplatin-based chemotherapy, but often develop resistance over time. With increasing use of carboplatin in the clinic, there is a pressing need to identify vulnerabilities of carboplatin-resistant tumors. In this study, we generated carboplatin-resistant TNBC MDA-MB-468 cell line and patient derived TNBC xenograft models. Mass spectrometry-based proteome profiling demonstrated that carboplatin resistance in TNBC is linked to drastic metabolism rewiring and upregulation of anti-oxidative response that supports cell replication by maintaining low levels of DNA damage in the presence of carboplatin. Carboplatin-resistant cells also exhibited dysregulation of the mitotic checkpoint. A kinome shRNA screen revealed that carboplatin-resistant cells are vulnerable to the depletion of the mitotic checkpoint regulators, whereas the checkpoint kinases *CHEK1* and *WEE1* are indispensable for the survival of carboplatin-resistant cells in the presence of carboplatin. We confirmed that pharmacological inhibition of CHEK1 by prexasertib in the presence of carboplatin is well tolerated by mice and suppresses the growth of carboplatin-resistant TNBC xenografts. Thus, abrogation of the mitotic checkpoint by CHEK1 inhibition re-sensitizes carboplatin-resistant TNBCs to carboplatin and represents a potential strategy for the treatment of carboplatin-resistant TNBCs.

## Introduction

Breast cancer has the highest estimated incidence rate across all cancers^1^. Triple negative breast cancer (TNBC) accounts for 12–17% (depending on immunohistochemical cut-offs used) of all breast cancer patients and are defined by the lack of expression of estrogen receptor (ER), progesterone receptor (PR), and human epidermal growth factor receptor 2 (HER2). This subgroup is more aggressive and is associated with an unfavorable prognosis^2,3^. Treatment of TNBCs depends on chemotherapy, due to the lack of specifically approved targeted therapies. The majority of patients with early stage TNBC are treated with neoadjuvant chemotherapy as pre-surgery treatment. This approach can enable breast-conserving surgery, provides an early exposure of micrometastatic disease to therapy, and tests the tumor’s sensitivity to chemotherapy.

Cisplatin chemotherapy has been in use to great effect for half a century for the treatment of different tumor types. In a bid to limit its toxicity, its derivative, carboplatin, was discovered over a decade later. Carboplatin shares cisplatin’s considerable efficacy, but has more acceptable adverse effects^4^. In 2014, two phase II clinical trials (GeparSixto, NCT01426880 and Alliance, NCT00861705) demonstrated that the addition of carboplatin to the standard anthracycline-taxane neoadjuvant regimens increased the pathological complete response (pCR) rate in TNBC patients^5–7^. Consistently, a recent study reported improvements in both disease-free and overall survival in patients receiving carboplatin^8^. The Brightness (NCT02032277)^9^ and I-SPY 2 (NCT01042379) trials confirmed that carboplatin increases the pCR rate of TNBC up to 50–55% from 30 to 35% for conventional chemotherapy. pCR to neoadjuvant chemotherapy, which includes carboplatin, increases disease-free survival in TNBC^10,11^, with only 5–6% experiencing distant recurrence. Patients with metastases or heavy tumor burden also seem to benefit from carboplatin treatment^12^.

Carboplatin contains a platinum atom complexed with two ammonia groups and a cyclobutane-dicarboxyl residue. Once the agent is intracellularly activated, it forms reactive platinum complexes that bind to nucleophilic groups such as GC-rich sites in DNA, inducing DNA cross-links that lead to double strand breaks (DSBs)^13^. Deficiencies in DNA repair pathways such as nucleotide excision DNA repair^14^ or homologous recombination DNA repair, such as loss-of-function mutations in the Breast Cancer Type 1 Susceptibility Protein (*BRCA1*) and *BRCA2* genes^15^ are associated with higher TNBC sensitivity to carboplatin treatment. This suggests that the combination of carboplatin with a targeted therapy, which impairs the DNA Damage response, increases its efficacy and potentially overcomes carboplatin resistance. However, such combinations, for example with the PARP-inhibitor veliparib^9^, have yet failed to prove clinical benefit in TNBC.

Platinum compounds also form adducts with mitochondrial DNA that impair the synthesis of electron transport chain proteins and induce a mitochondria-dependent reactive oxygen species (ROS) response. Besides DNA crosslinking, platinum drugs also react with RNA and proteins^13,16^. In particular, platinum compounds affect protein activity by forming adducts with functional protein groups, especially with cysteine and methionine side chains^13,16^. In summary, carboplatin cytotoxicity is mediated by multiple components whose relative contributions in causing cell death may depend on cell proliferation rate, DNA repair efficiency, redox status, and metabolic activity.

Although many TNBCs are initially susceptible to chemotherapy with platinum drugs^6,9^, over time they often develop resistance to carboplatin^16^. Resistance could be achieved by more efficient DNA damage repair, drug inactivation with glutathione and metallothioneins, increased drug efflux by membrane transport systems, upregulation of antioxidant and anti-proteotoxic responses, and induction of pro-survival signaling^13,16^. Furthermore, a large-scale genome analysis of chemo-resistant ovarian tumors also revealed frequent inactivation of tumor suppressors, such as retinoblastoma–associated protein (*RB1*), neurofibromin 1 (*NF1*), and phosphatase and tensin homolog (*PTEN*)^17^. These insights have guided attempts to improve the therapeutic efficacy of carboplatin treatment. For example, increased EGFR expression has been reported to confer platinum resistance in TNBCs by inducing pro-survival signaling^18^. However, the EGFR inhibitor cetuximab did not show any benefit in combination with carboplatin^19^. Other studies attempted to overcome resistance by increasing the amount of active platinum in the cell by targeting the multidrug resistance-protein 1 (MDR1). Unfortunately, tested MDR1 inhibitors also did not show a significant clinical benefit^20^. Considering the clinical potential of carboplatin, there is an urgent clinical need to understand how to overcome carboplatin resistance.

In this study, we found that carboplatin-resistant TNBCs depend on the mitotic checkpoint pathway to survive carboplatin treatment. Targeting this pathway could represent a major step in overcoming platinum resistance. Specifically, the inhibition of checkpoint kinase 1 (CHEK1) overcame carboplatin resistance both in vitro and in a pre-clinical carboplatin-resistant patient-derived tumor xenograft (PDX) model. The superior efficacy of carboplatin, when used in combination with CHEK1 inhibition, could broaden the target population of TNBC patients and overcome resistance in those patients who do not respond to the therapy.

## Results

### Carboplatin resistance in TNBCs leads to global proteome rewiring to resist carboplatin-induced oxidative and genotoxic stress

**Figure 1:**
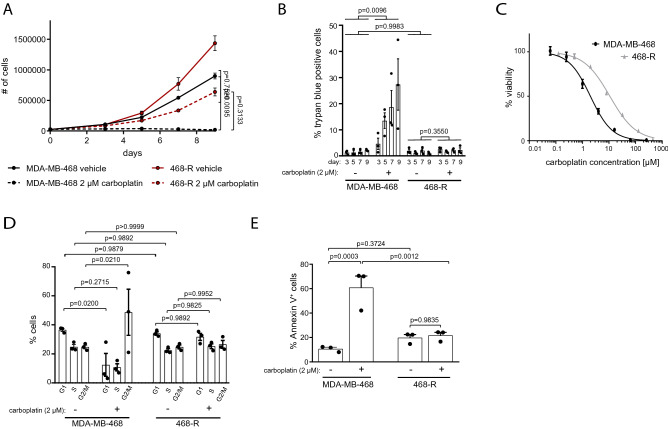
468-R cells can grow in the presence of carboplatin. (**A**,**B**) Cell growth (**A**) and cell death (**B**) of MDA-MB-468 and 468-R cells treated with vehicle or 2 µM carboplatin. Data are shown as Mean ± SEM. *P* values were calculated by two-way ANOVA with Geisser–Greenhouse and Tukey’s correction. (N = 3). (**C**) Dose–response after three days carboplatin treatment of MDA-MB-468 (N = 3) and 468-R (N = 6) cell lines. R^2^: MDA-MB-468 = 0.9799, 468-R = 0.9872. Data are shown as Mean ± SEM. (**D**,**E**) Cell cycle distributions and apoptosis analysis of MDA-MB-468 and 468-R cells treated for 5 days with vehicle or 2 µM carboplatin. Propidium iodide- (**D**) or Annexin V-stained (**E**) cells were analyzed by flow cytometry. Data are shown as Mean ± SEM. *P* values were calculated by two-way ANOVA with Geisser–Greenhouse and Tukey’s corrections. (N = 3).

To understand the mechanisms underlying carboplatin resistance, we generated a carboplatin-resistant model of TNBC. Consistent with its basal-like 1 phenotype, the TNBC cell line MDA-MB-468 showed high sensitivity to carboplatin treatment (Fig. 1A). By continuously exposing MDA-MB-468 cells for 10 months to gradually increased concentrations of carboplatin, we generated MDA-MB-468 resistant cells (468-R) that are able to proliferate in the presence of 2 µM carboplatin (Fig. 1A,B). The resistant cells showed a tendency to grow at higher rate compared to parental MDA-MB-468 cells even in the absence of carboplatin. Thus, the carboplatin resistance in our model could not be explained by decreased growth rate. The dose of carboplatin needed to inhibit cell growth by 50% (IC50) was more than five times higher for 468-R cells (10.75 µM) than parental MDA-MB-468 (2.00 µM) (Fig. 1C). Five days of incubation with carboplatin significantly impaired the growth of sensitive MDA-MB-468 cells, as detected by a reduced fraction of cells in G1 and S phases of the cell cycle and accumulation in the G2/M phase. On the other hand, carboplatin did not affect the cell cycle distribution of 468-R cells (Fig. 1D), indicating that 468-R cells are capable of proliferating in the presence of carboplatin. Furthermore, five days of carboplatin treatment led to apoptosis of parental MDA-MB-468 cells, but not of resistant 468-R cells as detected by Annexin V staining (Fig. 1E).

**Figure 2:**
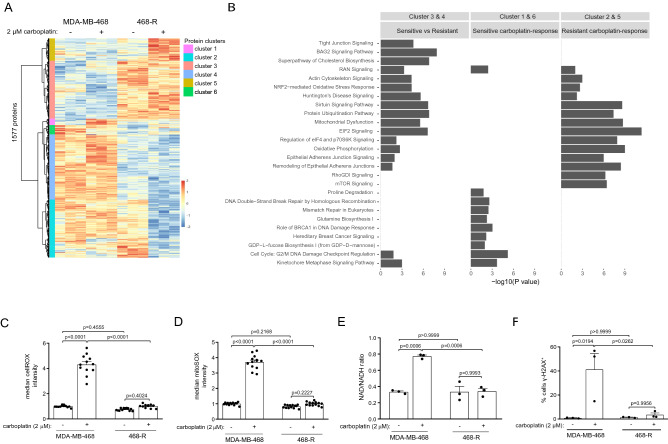
Proteome re-wiring of carboplatin-resistant 468-R cells protects from carboplatin-induced oxidative and genotoxic stress. (**A,B**) Hierarchical clustering of differentially expressed proteins (DE) (**A**) and Ingenuity Pathway Analysis (**B**) of the proteome alterations in MDA-MB-468 and 468-R cells treated with vehicle or 2 µM carboplatin for 5 days. Protein expression was analyzed by LC/MS–MS, and DE proteins were identified by ANOVA followed by Tukey’s HSD test with an adjusted *P* value < 0.05. The top 10 significantly enriched pathways are shown. *P* value < 0.05 was used to determine significantly enriched pathways; the –log10 (*P* value) was not shown if the pathway was not significantly enriched. N = 3. (**C,D**) ROS levels in MDA-MB-468 and 468-R cells treated with vehicle or 2 µM carboplatin for 5 days. Total ROS levels (**C**) were measured by the cellROX assay, and mitochondrial specific superoxide levels (**D**) were measured by the mitoSOX assay. Median flow of cytometric intensities were normalized to MDA-MB-468 vehicle condition. Data are shown as Mean ± SEM. *P* values were calculated by two-way ANOVA with Geisser–Greenhouse and Tukey’s correction. N = 3. (**E**) NAD/NADH ratio as measured by NAD/NADH-Glo kit. Data are shown as Mean ± SEM. *P* values were calculated by two-way ANOVA with Geisser–Greenhouse and Tukey’s correction. N = 3. (**F**) γ-H2AX staining of MDA-MB-468 and 468-R cells treated with vehicle or 2 µM carboplatin for 5 days as analyzed by flow cytometry. *P* values were determined by two-way ANOVA with Geisser–Greenhouse and Tukey’s corrections. N = 3. Data are shown as Mean ± SEM.

To elucidate the underlying mechanics of carboplatin resistance, we performed proteome profiling of parental MDA-MB-468 and carboplatin-resistant 468-R cells in the presence or absence of 2 µM carboplatin. To interrogate the differences between parental and resistant TNBC cells, we performed unsupervised hierarchical clustering of differentially expressed proteins in MDA-MB-468 and 468-R cells. Carboplatin resistance was associated with a global proteome re-wiring of carboplatin-resistant cells. We identified 6 clusters of proteins with similar expression patterns, highlighting differences between parental and resistant cells (Cluster 3 and 4), or linked to response to carboplatin treatment in parental (Cluster 1 and 6) and resistant cells (Cluster 2 and 5) (Fig. 2A).

We next performed an enriched pathway analysis of these protein clusters using Ingenuity Pathway Analysis (IPA) (Fig. 2B). The pathway analysis revealed that carboplatin resistance was accompanied with a rewiring of the translation-related pathways, such as EIF2 Signaling and Regulation of eIF4 and p70S6K. Carboplatin-resistant cells also exhibited several metabolic alterations, affecting the Sirtuin Signaling Pathway, Mitochondrial Dysfunction, and Oxidative Phosphorylation (Fig. 2A,B). Strikingly, the same pathways were significantly altered in response to carboplatin treatment in the resistant cells, highlighting the contribution of these pathways to carboplatin resistance. These pathways not only allow adaptation to carboplatin-induced oxidative stress^21–23^, but also up-regulate nucleotide biosynthesis^21–23^, a process crucial to coping with DNA damage, while maintaining proliferation^24–26^. Concordantly with the proteome analysis, we detected dramatically lower levels of ROS, mitochondrial-specific superoxide as well as a lower ratio of oxidized Nicotinamide Adenine Dinucleotide (NAD) to reduced NADH in carboplatin-treated 468-R cells compared to sensitive carboplatin-treated MDA-MB-468 cells (Fig. 2C-E).

Metabolic rewiring and low ROS levels in carboplatin-resistant cells could explain similar levels of DSBs in untreated and carboplatin-treated 468-R cells, as detected by flow cytometry analysis and immunocytochemistry of phosphorylated histone H2AX (γ-H2AX) (Fig. 2F, Supplementary Figure S1A,B). We also did not find any alterations in the DNA damage or checkpoint pathways upon carboplatin treatment of 468-R cells (Fig. 2A,B). In contrast, carboplatin treatment of carboplatin-sensitive TNBC cells led to DNA damage and activation of the DNA damage pathways, such as DNA Double − Strand Break Repair by Homologous Recombination, Mismatch Repair in Eukaryotes, and Role of BRCA1 in DNA Damage Response, as well as the checkpoint pathways, Cell Cycle: G2/M DNA damage Checkpoint Regulation and Kinetochore Metaphase Signaling Pathway (Fig. 2B). Altogether, these data indicate that acquisition of carboplatin resistance in TNBC is linked to drastic proteome rewiring, leading to alterations in metabolism and upregulation of anti-oxidative stress responses to overcome DNA damage and support cell proliferation.

### The mitotic checkpoint is essential for survival of carboplatin-resistant cells

**Figure 3:**
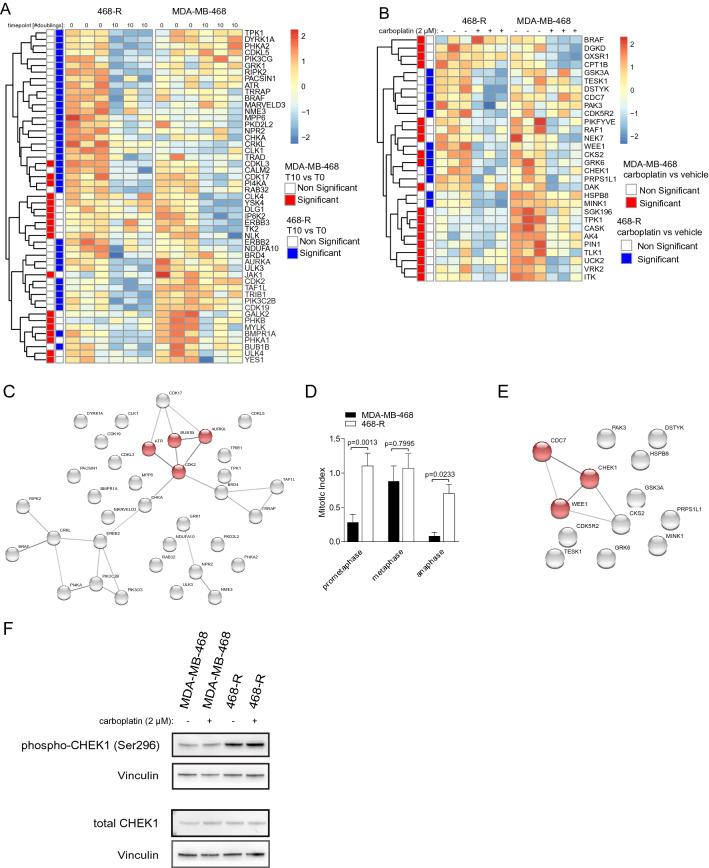
A pooled shRNA kinome screen to identify the vulnerabilities of carboplatin-resistant cells. A pooled shRNA kinome library was transduced into MDA-MB-468 and 468-R cells, and the cells were cultured in the absence or presence of carboplatin (0.3 µM for MDA-MB-468 cells or 2.0 µM for 468-R cells) for more than 10 doublings. (**A,B**) Heatmaps of gene-scores for genes whose targeting shRNAs were significantly depleted at the end of the shRNAi screen in the absence (**A**) or presence of carboplatin (**B**). Gene-scores were compared by ANOVA followed by Tukey’s HSD test. Significant genes (adjusted *P* value < 0.1) are indicated in red for MDA-MB-468 or in blue for 468-R cells. (**C**) The String pathway analysis of essential genes for 468-R cells. The regulators of the mitotic cycle are shown in red. (**D**) Mitotic index in DAPI-stained MDA-MB-468 (N = 2221) and 468-R (N = 4537) cells. Data are shown as Mean ± SEM. *P* values were determined by two-way ANOVA with Geisser–Greenhouse and Sidak’s corrections. (**E**) The String pathway analysis of carboplatin-survival genes for 468-R cells. The components of the G2/M Checkpoint are shown in red. (**F**) Immunoblot analysis of MDA-B-468 and 468-R cells treated for five days with vehicle or 2 μM carboplatin with the indicated antibodies. Uncropped blots are presented in Supplementary Figure S4.

To identify the vulnerabilities of carboplatin-resistant TNBC, we performed a kinome shRNA screen. We expressed 12 shRNAs, on average, for each of 752 kinases or kinase-related genes (9421 shRNAs in total) in carboplatin-sensitive parental MDA-MB-468 cells and their carboplatin-resistant 468-R counterpart. After puromycin selection (refers as T0), the cells were exposed to either vehicle, or carboplatin at a concentration that inhibited their growth by 70%; 0.3 µM for MDA-MB-468 cells and 2.0 µM for 468-R cells for more than 10 doublings (T10 vehicle or T10 carboplatin) (Fig. 3A). To identify genes, for which targeting shRNAs showed overall depletion, we calculated a score for each gene using normalized shRNA expression. Using the ANOVA method, we identified essential genes by comparing T0 and T10 scores, whereas the comparison of gene scores in T10 vehicle and T10 carboplatin identified carboplatin-survival genes. Consistent with the global proteome rewiring of carboplatin-resistant cells, we observed only a partial overlap between the genes identified for parental MDA-MB-468 and resistant 468-R cells (Fig. 3A,B).

String pathway analysis of essential genes for 468-R cells revealed a dependency on the regulators of the mitotic cycle, Aurora Kinase A *(AURKA)*, BUB1 Mitotic Checkpoint Serine/Threonine Kinase B *(BUBR1)*, and Cyclin Dependent Kinase 2 *(CDK2)* (‘Regulation of Mitotic Cell Cycle’ FDR = 0.026) (Fig. 3C). This observation is in line with carboplatin-induced alterations in the Kinetochore Metaphase Signaling Pathway (Fig. 2B) and longer prometaphase and anaphase in carboplatin-resistant 468-R cells compared to the parental MDA-MB-468 cells (Fig. 3D). These results are consistent with a previous observation showing mitotic exit vulnerability driven by Anaphase Promoting Complex/Cyclosome (APC/C) dysfunction in several cisplatin-resistant models of epithelial ovarian cancer^27^.

On the other hand, String pathway analysis of carboplatin-survival genes for 468-R cells showed enrichment of the G2/M checkpoint components, G2 Checkpoint Kinase *WEE1,* Cell Division Cycle 7 (*CDC7*), and *CHEK1* (Fig. 3E). The G2/M checkpoint pathway regulates the cell cycle in response to replication stress and DNA damage, including the DNA-crosslinks caused by platinum agents^28^. The kinase CHEK1, a major component of the pathway, facilitates the continuation of cell division by stabilizing the replication fork and activating DNA damage repair mechanisms^26^. The WEE1 kinase, downstream of CHEK1, is a CDK antagonist that slows the cell cycle to adapt to genotoxic stress^29–31^. Even though platinum agents are well known to induce DNA damage and replication stress, carboplatin-treated 468-R cells exhibited low levels of DNA damage. Moreover, we found similar cell cycle distributions and comparable levels of 5-Ethynyl-2´-deoxyuridine (EdU)-incorporation in 468-R in the absence or presence of carboplatin (Fig. 1D, Supplemental Figure S1C). This suggests that depletion of *CHEK1* or *WEE1* re-sensitizes 468-R cells to carboplatin by mechanisms other than abrogation of the G2/M checkpoint control.

Another well-described function of CHEK1 is its role in the maintenance of spindle checkpoint function^30^, delaying anaphase until proper alignment of chromosomes^31^. CHEK1 is also required for stable metaphase arrest^32^, whereas CHEK1 deficient cells show decreased localization of the checkpoint kinase BUBR1 to kinetochores^30^. WEE1-mediated inhibition of CDK1 could block mitotic entry, whereas CDC7 kinase was reported to stimulate Aurora B kinase activity during mitosis^33,34^. Given that we observed mitotic checkpoint aberration in carboplatin-resistant 468-R cells (Figs. 2B, 3C), this suggests that abrogation of mitotic control through blockade of WEE1 or CHEK1 might induce death of carboplatin-treated 468-R cells. Moreover, we found increased levels of CHEK1 activity in carboplatin-resistant 468-R cells compared to sensitive parental cells, further confirming the dependency of carboplatin-resistant cells to CHEK1 activity (Fig. 3F). In summary, our results indicate that targeting of the mitotic checkpoint could re-sensitize carboplatin-resistant TNBCs to carboplatin.

### Pharmacological inhibition of CHEK1 or WEE1 re-sensitizes carboplatin-resistant cells

**Figure 4:**
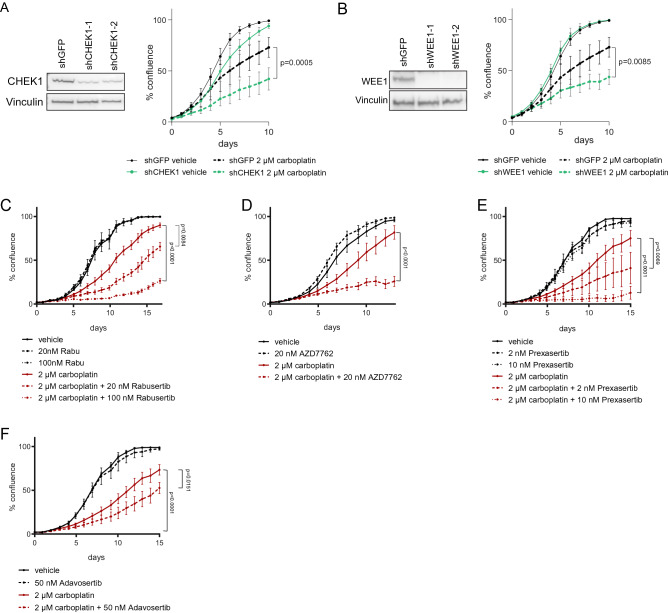
Inhibition of CHEK1 and WEE1 re-sensitize carboplatin-resistant 468-R cells. The growth of 468-R cells was analyzed by an Incucyte imaging system. (**A,B**) *CHEK1* or *WEE1* knockdown in 468-R cells expressing shRNAs against *GFP*, *CHEK1* or *WEE1* were confirmed by immunoblotting (left). Uncropped blots are shown in Supplementary Figure S4. The growth of the indicated cells treated with vehicle or 2 µM carboplatin for 10 days (right). Data are shown as the mean of two independent shRNAs targeting *CHEK1* or *WEE1*. *P* values were calculated by two-way ANOVA with Geisser–Greenhouse and Dunnett’s corrections. Data are shown as Mean ± SEM. N = 7. (**C–F**) 468-R cells treated for up to 17 days with 2 µM carboplatin alone or in combination with CHEK1 inhibitor rabusertib (N = 3) (**C**), CHEK1/2 inhibitor AZD7762 (N = 4) (**D**), CHEK1 inhibitor prexasertib (N = 5) (**E**), or WEE1 inhibitor adavosertib (N = 5) (**F**). *P* values were calculated by two-way ANOVA with Geisser–Greenhouse and Dunnett’s corrections. Data are shown as Mean ± SEM.

To validate the results of the shRNA screen, we assessed the growth of carboplatin-resistant 468-R cells after suppression of *CHEK1* or *WEE1* by two independent short hairpins. In agreement with the screen results, we found that depletion of either *CHEK1* or *WEE1* inhibited the growth of carboplatin-treated 468-R cells, confirming the dependence of carboplatin-resistant cells on these checkpoint kinases (Fig. 4A,B).

Next, we assessed the efficiency of pharmacological inhibition of CHEK1 or WEE1 in re-sensitization of carboplatin-resistant cells to carboplatin treatment. We chose several CHEK1 and WEE1 inhibitors, for which clinical data are available. AZD7762, a CHEK1/CHEK2 inhibitor, showed a complete response in a patient with metastatic small-cell renal cancer when combined with irinotecan^35^. The more selective CHEK1 inhibitor rabusertib was well tolerated in combination with gemcitabine^36^. Prexasertib, an even more selective CHEK1 inhibitor, has been tested as monotherapy^37^. It showed only transient adverse events, and was able to induce modest responses as monotherapy in patients with advanced cancer^37,38^. For WEE1 inhibition, we selected adavosertib as it was shown to effectively inhibit WEE1 activity in advanced cancer patients^39^, and the combination of adavosertib with carboplatin has already shown encouraging results in treating carboplatin-resistant ovarian cancer^40^.

We tested the ability of the selected inhibitors to inhibit growth of carboplatin-resistant 468-R cells. As a single agent, all tested CHEK1 and WEE1 inhibitors impaired the growth of 468-R cells only at the highest concentrations or not at all (Supplemental Figure 2A–C). On the other hand, when combined with even low doses of carboplatin all CHEK1/WEE1 inhibitors re-sensitized carboplatin-resistant 468-R cells (Fig. 4C–F). These results indicate that inhibitors of CHEK1 or WEE1 in combination with carboplatin could represent an effective strategy for the treatment of carboplatin-resistant TNBCs.

### Low dose prexasertib is non-toxic in combination with carboplatin

**Figure 5:**
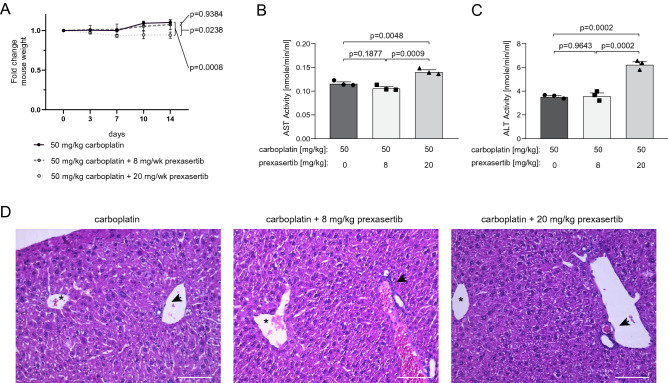
A low dose of prexasertib in combination with carboplatin is well tolerated by mice. Healthy NMRI nude mice treated weekly with carboplatin (50 mg/kg) in combination with either vehicle, low dose (8 mg/kg) or high dose (20 mg/kg) of Prexasertib for two weeks (N = 3). (**A**) Relative weight of the mice. Data are shown as Mean ± SEM. *P* values were determined by two-way ANOVA with Geisser–Greenhouse and Tukey’s corrections. (**B,C**) serum activity of the AST (**B**) and ALT (**C**) liver enzymes. Data are shown as Mean ± SEM. *P* values were determined by ordinary one-way ANOVA with Tukey’s correction. (**D**) H&E stainings showing livers of mice treated with the indicated combinations. The images show a terminal vein surrounded by normal liver architecture consisting of plates of hepatocytes one cell thick, separated by normal-looking sinusoids. No microabscesses, nor confluent areas of necrosis or inflammation were noticed around the terminal vein, nor around the portal spaces. Only sporadic isolated apoptotic hepatocytes were observed in all the specimens, and not in relation to a particular functional zone of the liver parenchyma. Asterisks denote terminal veins. Arrowheads denote portal tracts. Scale bar, 100 µm.

Even though all tested CHEK1 or WEE1 inhibitors suppressed the growth of carboplatin-resistant cells in combination with carboplatin, for in vivo validations we decided to focus on prexasertib, which is currently in phase II of clinical trials. First, we assessed the toxicity of the combinational treatment of carboplatin and prexasertib compared to carboplatin alone. We treated NMRI nude mice with either a low (8 mg/kg) or a high (20 mg/kg) weekly dose of prexasertib in combination with 50 mg/kg carboplatin. We found that the higher dose of prexasertib led to weight loss and yellow skin rash in some mice, whereas we did not observe this in mice treated with the low dose regimen (Fig. 5A).

To assess whether the combinational treatment induces liver injury, we measured the activity of aspartate- (AST) and alanine (ALT) aminotransferases in serum of the treated mice. The activity of both liver enzymes was not increased in the serum of mice treated with the low dose of prexasertib in combination with carboplatin (Fig. 5B,C). Furthermore, standard histological analysis did not reveal prominent changes in the liver of mice following the administration of the combinational treatments as compared with those of mice in the carboplatin-treated group (Fig. 5D). Livers from all mice did show scattered apoptotic or mitotic hepatocytes, probably resulting from carboplatin treatment. The presence of megakaryocytes in the spleens of these mice was also unaffected, which could indicate sustained thrombopoiesis. Moreover, we did not find treatment-related abnormalities in kidney, pancreas, heart and lung (Supplemental Figure S3). Thus, a combination of a low dose of prexasertib with carboplatin is well tolerated by mice.

### CHEK1 inhibition sensitizes carboplatin-resistant TNBC to carboplatin in vivo

**Figure 6:**
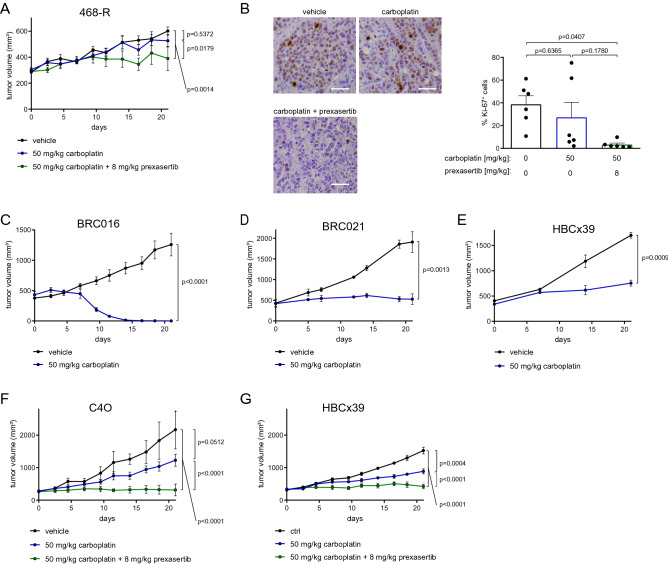
A low dose of prexasertib in combination with carboplatin overcomes platinum resistance in TNBC xenograft models. (**A,B**) tumor growth (**A**) *and Ki67 positivity* (**B**) of 468-R TNBC cell line xenografts treated with vehicle (N = 6), 50 mg/kg carboplatin (N = 7), or 50 mg/kg carboplatin and 8 mg/kg Prexasertib (N = 6) for three weeks. Scale bar, 50 µm. Data are shown as Mean ± SEM. *P* values were determined by two-way ANOVA with Geisser–Greenhouse and Tukey’s corrections, N = 6 for each condition. (**C–E**) The indicated patient-derived TNBC xenografts were treated with vehicle or 50 mg/kg carboplatin for 3 weeks. Tumor volumes of BRC016 (N = 9) (**C**), BRC021 (vehicle, n = 3; carboplatin n = 2) (**D**) and HBCx39 (N = 3) (**E**) PDX models are shown as Mean ± SEM. *P* values were determined by two-way ANOVA with Geisser–Greenhouse correction. (**F,G**) The growth of C4O (N = 3 per condition) (**F**) and HBCx39 (N = 6 vehicle-treated; N = 6 carboplatin-treated; N = 7 carboplatin + prexasertib-treated) (**G**) PDX xenografts. PDX tumors were treated with vehicle, 50 mg/kg carboplatin, or 50 mg/kg carboplatin and 8 mg/kg Prexasertib, for 3 weeks. Data are shown as Mean ± SEM. *P* values were determined by two-way ANOVA with Geisser–Greenhouse and Tukey’s corrections.

We next assessed the efficiency of the combinational treatment of carboplatin and prexasertib in vivo. Consistent with the results of the in vitro studies, we found that carboplatin did not affect the growth rate of the 468-R xenograft tumors. In contrast, a low dose of prexasertib in combination with carboplatin suppressed 468-R xenograft growth (Fig. 6A). CHEK1 inhibition in the presence of carboplatin also led to decreased number of Ki-67-positive cells (Fig. 6B).

As PDX models better reflect patient’s responses than cell line models^41^, we tested the carboplatin sensitivity of three PDX models, established from triple negative primary invasive ductal adenocarcinomas. The PDX model, BRC016, derived from a chemotherapy-naïve tumor, showed high sensitivity to carboplatin, achieving a complete response to carboplatin in nine out of ten mice (Fig. 6C). Two other models were generated from two tumors that received non-platinum neoadjuvant chemotherapy consisting of epirubicin, cyclophosphamide, and taxane. The PDX model BRC021 showed only partial response to carboplatin, maintaining a stable disease (Fig. 6D), whereas the HBCx39 xenograft was able to grow slowly in the presence of carboplatin (Fig. 6E). The latter observation is consistent with the poor tumor response when the patient underwent the first line of adjuvant carboplatin. This variability in carboplatin sensitivity of different PDX models reflected the differences in response observed in TNBC patients.

Remarkably, an individual xenograft tumor of the sensitive BRC016 model achieved only a partial response to carboplatin and even resisted a second line of carboplatin. We used this tumor to establish a novel carboplatin-resistant PDX model, C4O (Fig. 6F). We found that carboplatin did not inhibit the growth of the C4O xenograft. On the other hand, the combination of carboplatin with a low dose of prexasertib suppressed the growth of this carboplatin-resistant model, resulting in a stable disease (Fig. 6F). Similarly, while carboplatin reduced the growth of the second carboplatin-resistant PDX model, HBCx39 minimally, the combination of carboplatin with low dose prexasertib halted growth almost entirely (Fig. 6G). These results confirmed the efficacy of the combination of prexasertib and carboplatin to treat carboplatin-resistant TNBCs.

## Discussion

In this preclinical study, we developed new in vitro and patient-derived models of carboplatin resistance in TNBC. By employing unbiased proteomic and shRNA screens, we uncovered a targetable dependency of carboplatin-resistant TNBC on the mitotic checkpoint. The combination of carboplatin with pharmacological inhibitors of CHEK1 or WEE1 inhibits the growth of carboplatin-resistant TNBCs, including patient-derived resistant tumors.

We found carboplatin-resistant TNBC cells show longer prometaphase and anaphase as well as alterations in the Kinetochore Metaphase Signaling Pathway. The mitotic checkpoint delays mitosis by inhibiting the APC/C complex. In cancer cells, mitotic control is commonly dysregulated to allow excess proliferation, whereas abrogation of this checkpoint could lead to mitotic catastrophe, senescence, or apoptosis^42^. The effect of platinum agents on the mitotic checkpoint is not well understood. A recent report has linked platinum resistance in ovarian cancer to APC/C dysfunction coupled with functional dependency on Polo-like kinase 1 (PLK1) for mitotic exit. Concordantly, we found that the mitotic checkpoint kinases, *BUBR1* and *AURKA*, are essential for the growth of carboplatin-resistant TNBC, implying an increased dependence of carboplatin-resistant cells on the mitotic checkpoint. Importantly, targeted therapies of the mitotic regulators such as Aurora A were recently designed to assault cancer-associated alterations in the mitotic checkpoint. The clinical response of unselected cancers to Aurora A inhibitors has been underwhelming^43^, even though we have not tested the effect of Aurora A inhibitors on the survival of carboplatin-resistant cells.

Our data also indicate synthetic lethality of carboplatin with the G2/M checkpoint kinases, CHEK1 and WEE1 in TNBC. Whereas carboplatin’s genotoxicity and oxidative assault^13,16^ are well described to induce DNA damage and replication stress^28^, we found only a slight increase in oxidative stress and DNA damage, and no evidence of replication stress in response to carboplatin treatment in our carboplatin-resistant TNBC model, suggesting that depletion of CHEK1 or WEE1 re-sensitizes 468-R cells to carboplatin by mechanisms other than abrogation of G2/M checkpoint control. Given that the G2/M and mitotic checkpoints are tightly intertwined^32^, this suggests that loss of CHEK1 or WEE1 suppresses the growth of carboplatin-treated 468-R cells due to abrogation of the mitotic checkpoint.

Our results also indicate that pharmacological inhibition of WEE1 or CHEK1 re-sensitizes carboplatin-resistant TNBC to carboplatin. In current clinical trials, the CHEK1 inhibitor prexasertib showed least toxicities and the most encouraging results as monotherapy^37^. Even so, its effect was modest (stable disease 33.3%; partial response 4.4%) in the treatment of advanced cancers, similar to the single agent efficiency of other CHEK inhibitors, seemingly implying an inherent resistance^37,38^. In our preclinical models, prexasertib alone also showed limited efficacy, whereas the combination of prexasertib with carboplatin achieved a superior response, even with a low dose of prexasertib.

The compounded toxicities of prexasertib and carboplatin could be a cause for concern. Both prexasertib^38^ and carboplatin^5^ induce toxic haematological effects, in particular, neutropenia and thrombocytopenia, as their major adverse events. Nonetheless, the low dose of prexasertib that we used in our study in combination with an average dose of carboplatin induced significant tumor inhibition in vivo, without signs of toxicity. In vitro, our doses are below the concentrations measured in the serum of patients treated with the clinically recommended phase II dose for prexasertib^37^ or a standard infusion of carboplatin^44^, suggesting they could be used at lower clinical doses without losing efficacy. Pending a tolerable dose, the combination treatment could be used for both inherent and acquired resistant TNBC with or without selection for basal biomarkers, such as expression of high molecular weight cytokeratins and EGFR1 overexpression. This combination could increase the fraction of patients achieving the benefits of responders to carboplatin in the neoadjuvant^5,6^, adjuvant^45^, and metastatic^46^ settings. In conclusion, we show that low dose prexasertib can sensitize carboplatin-resistant TNBC to carboplatin. We believe that this combination could potentially bring the remarkable benefits of responders to carboplatin^8,47,48^, to the majority of basal-like TNBC, providing more options to these otherwise difficult-to-treat tumors.

## Material and methods

### Cell lines and cell culture

MDA-MB-468 cells (ATCC HTB-132) were cultured in DMEM (Gibco), with 10% fetal bovine serum (FBS; Thermofisher), 100 µg/mL PenStrep (Gibco) and 2 mM L-glutamine (Gibco). Cells were tested with a mycoplasma testing kit (Lonza MycoAlert, LT07-318).

To induce resistance in MDA-MB-468 cells, the cells were exposed continuously to carboplatin (Hospira UK, Ltd), except for 48 h after splitting. The starting concentration of carboplatin was 0.4 µM and it was increased incrementally (nine times) until the cells grew comfortably at 2 µM carboplatin.

Lentiviral infections were performed as described by the RNAi Consortium (TRC). Infected cells were selected by puromycin selection (2 µg/mL; Thermofisher A11138-03).

### Analysis of cell growth, cell death and drug sensitivity

For growth inhibition, cell proliferation and cell death experiments, 25,000 cells were seeded in 12-well microplate (Greiner, 665180) wells and counted manually on a Neubauer hemocytometer with trypan blue dye exclusion. Proliferation curves of genetic and pharmacological Chk1 and Wee1 inhibition were generated using an IncuCyte ZOOM system (Essen BioScience) on 2000 cells seeded on 96-well microplate (TPP, 92696) wells, based on phase contrast images taken at 24 h intervals. Prexasertib (LY2606368) and adavosertib (AZD1775; MK-1775) were purchased from MedChemExpress (HY-18174 and HY-10993, respectively).

### Immunoblotting and Immunocytochemistry

Proteins from cells, lysed in Pierce RIPA buffer (Thermofisher) containing phosphatase and protease inhibitors (PhosSTOP and Complete, Roche), were quantified using Pierce BCA. Protein separation and blotting were performed on Mini-Protean TGX Precast gels (BioRad) and Mini Trans-Blot nitrocellulose membranes (BioRad), respectively. Membranes were incubated with primary antibody (Vinculin: mouse monoclonal 1:10,000, Sigma Aldrich V9131; CHEK1 (2G1D5): mouse monoclonal, Cell Signaling Technology, 2360; phospho-CHEK1 (133D3)(Ser296): rabbit monoclonal, Cell Signaling Technology 2348; WEE1 (B-11): mouse monoclonal, Santa Cruz sc-2585) overnight at 4 °C, and secondary antibody (1:5000 peroxidase-conjugated polyclonal goat anti-rabbit antibody, or 1:10,000 peroxidase-conjugated polyclonal goat anti-mouse antibody, Jackson ImmunoResearch, 111-035-003, and 115-035-003, respectively) for 1 h at room temperature. An Azure c600 detection system (Azure Biosystems) and SuperSignal West Dura Extended Duration Substrate (Thermofisher) were used for visualization.

Cells, grown on sterile Menzel coverslips (Thermofisher), were fixed with 4% paraformaldehyde in PBS for 10 min, then washed several times with PBS. For γ-H2AX immunostaining, cells were permeabilized with 100% methanol at − 20 °C for 10 min and blocked with 1% bovine serum albumin (BSA), 0.3% Triton X100 and 5% donkey serum (Sigma Aldrich, D9663) in PBS for 30 min at room temperature, incubated with anti-γ-H2AX mouse monoclonal antibody (clone JBW301; Millipore, 05-636) and donkey anti-mouse Alexa Fluor 488 secondary antibody (Thermofisher, A-21202). Coverslips were mounted using ProLong Gold Antifade Mountant with DAPI (Thermofisher, P36935). Images were taken using a Leica DM5500 widefield upright microscope. Quantifications were performed by manual counting of γ-H2AX puncta per nucleus.

### Flow cytometry

For analysis of apoptosis, the Alexa Fluor-488 annexin V/Dead Cell Apoptosis Kit was used according to the manufacturer’s protocol (Thermofisher). Briefly, cells were trypsinized, washed in PBS, resuspended in binding buffer and incubated with Annexin V tagged with Alexa Fluor 488.

For cell cycle analysis, the Click-iT EdU Alexa Fluor-647 Flow Cytometry Assay Kit was used as recommended by manufacturer (Thermofisher). Cells were harvested after 2.5 h incubation with 10 µM EdU, washed in 1% PBS-BSA and fixed in 4% PFA. For EdU detection, cells were incubated with the Alexa Fluor 647-dye azide diluted in reaction buffer according to manufacturer’s protocol. DNA staining was performed in 1% PBS-BSA supplemented with 1 µg/ml FxCycle Violet (Thermofisher).

To detect γ-H2AX levels, cells were permeabilized and stained with γ-H2AX-FITC antibody (mouse monoclonal antibody (CR55T33), Thermofisher, 53-9865-82) in 1% PBS-BSA for 1 h at room temperature, and washed with 1% PBS-BSA. Raw data were acquired with the MACSQuant VYB flow cytometer (Miltenyi Biotec) and analyzed using FlowJo software (BD).

Total ROS and mitochondrial superoxide were measured by CellROX Deep Red Reagent (Thermofisher, C10422r) and MitoSOX (Thermofisher, M36008) using BD FACSCanto II, BD Biosciences in the APC/A and PE-A channels, respectively, according to the manufacturer’s protocols.

### Detection of NAD + /NADH ratio

NAD^+^ and NADH levels were determined using NAD/NADH-glo assay (Promega, G9071) according to supplier’s instructions. A Victor X4 plate reader (Perkin Elmer) was used to measure bioluminescence.

### shRNA screen

The kinome shRNAi library was supplied by Sigma Aldrich (Merck Millipore). 468-R cells were transduced at a multiplicity of infection of 1 to minimize the incorporation of multiple shRNAs per cell. After puromycin selection (2 µg/mL; Thermofisher A11138-03), cells were either collected (T0 control), or grown for over 10 doublings in the absence (T10 control) or presence of 2 µM carboplatin (T10 carboplatin). Cell pellets were shipped to Sigma Aldrich (Merck Millipore) for DNA sequencing and data deconvolution (Sigma Aldrich CSTSEQ).

The shRNA raw read counts were normalized to reads per million (RPM) for each sample, then transformed by log2 (RPM + 1). To identify genes, for which specific shRNAs were overall depleted, a gene-score was computed by R package (GSVA). The GSVA is a non-parametric and unsupervised algorithm that estimates pathway activity using gene expressions, transforming the gene expression matrix to a pathway enrichment score matrix. For shRNA screen analysis, targeting genes were treated as ‘pathways’ and shRNAs as ‘genes’. The GSVA was applied to shRNAs that targeted the same gene to summarize normalized expressions into a single score. The significance of gene-scores between T0, T10 vehicle and T10 carboplatin were determined by ANOVA followed by Tukey’s HSD Test with an adjusted *P* value < 0.1. Significant genes were used as an input for canonical pathway analysis by the String pathway analysis.

### Liquid chromatography–tandem-mass spectrometry (LC–MS/MS)

Cell-pellets of MDA-MB-468 and 468-R cells, treated for five days with either vehicle or 2 µM carboplatin were analyzed by Liquid chromatography–tandem-mass spectrometry (LC–MS/MS) as described in the Supplemental Methods.

The mass spectrometry proteomics data were deposited to the ProteomeXchange Consortium via the PRIDE partner repository with the dataset identifier PXD021726 (login username: reviewer_pxd021726@ebi.ac.uk and password: 9uD57dKL).

### 468-R Xenografts and PDX models

All experimental procedures were performed in accordance with relevant guidelines and regulations. The tumor tissue for implantation of PDX was acquired from a patient who had provided written informed consent and the procedure was approved by the Commission of Medical Ethics of the University Hospitals Leuven (approval numbers S54185 and ML8713). All animal experiments were approved by the Ethics Committee Research UZ/KU Leuven (approval number P038/2015).

3 × 10^6^ 468-R cells in 50% matrigel (Corning, Matrigel Basement Membrane Matrix, Growth Factor Reduced, 734-1100) were injected into the right flank of 20 nude mice (NMRI-Fox1nu strain, Taconic).

For Ki-67 immunohistochemistry, tissue, fixed in 4% formalin, was sectioned (4 µm), dewaxed with xylene and rehydrated in a series of ethanol. Antigen-retrieval was performed using a Tris/EDTA (pH 9.0) solution (Aviva Systems Biology OOMB00007) at 100 °C for 20 min. Endogenous peroxidases were blocked with 0.1% hydrogen peroxide in methanol for 20 min at room temperature. After washing (Tris buffer, pH 7.9), slides were incubated with primary Ki-67 rabbit monoclonal antibody (Thermofisher, RM-9106-R7) overnight at 4 °C and 1 h with peroxidase-conjugated polyclonal goat anti-rabbit antibody (Jackson ImmunoResearch, 111–035-003) at room temperature. We used the chromogen 3,3′-diaminobenzidine revelation kit (VECTOR, SK-4100) without nickel according to manufacturer’s instructions. The percentage of positive cells was counted manually (in > 500 cells) in at least five different tumors per condition.

BRC016 and BRC021 PDX models were established at the University Hospital UZ Leuven and HBCx39, established previously^49^, was obtained in collaboration with the Institut Curie. Treatment experiments included 25–50 nude mice (NMRI-Fox1nu strain, Taconic), implanted with TNBC tumor fragments. When tumors reached a volume of ~ 300 mm^3^ mice were randomly assigned to carboplatin (50 mg/kg) or vehicle (0.9% saline) cohorts. Mice were injected intraperitoneally once a week for 3 weeks before sacrifice. For prexasertib treatment, mice were injected twice, with an 8-h interval, subcutaneously on days one and four of a seven-day cycle. Length (L) and width (W) of tumors were measured with a caliper and the volume was estimated as V = L × W^2^ × π/6.

For the toxicity study, healthy 16-week-old NMRI nude mice were treated for two weeks with 50 mg/kg carboplatin plus either vehicle, 8, or 20 mg/kg prexasertib per week. Necropsy of all organs was performed and liver, lung, heart, spleen, kidneys, and pancreas were collected for H&E staining.

## Supplementary Information

Supplementary Information.
