# Supplementary material for: The mitotic checkpoint is a targetable vulnerability of carboplatin-resistant triple negative breast cancers
Source: Sci Rep. 2021 Feb 4;11:3176. doi: 10.1038/s41598-021-82780-6 (PMC7862668; doi:10.1038/s41598-021-82780-6)
Supplement: Supplementary file 1 — Supplementary Information. [file 41598_2021_82780_MOESM1_ESM.pdf]

Targeting the mitotic checkpoint re-sensitizes carboplatin-resistant triple negative breast cancers

Stijn Moens[1,2], Peihua Zhao[1,2], Maria Francesca Baietti[1,2], Oliviero Marinelli[2,3], Delphi Van Haver[4,5,6], Francis Impens[4,5,6], Giuseppe Floris[7,8], Elisabetta Marangoni[9], Patrick Neven[2,10], Daniela Annibali[2,11*], Anna A Sablina[1,2,*], Frédéric Amant[2,10,12,*]

VIB-KU Leuven Center for Cancer Biology, VIB, Belgium[1]; Department of Oncology, KU Leuven and Leuven Cancer Institute (LKI), 3000 Leuven, Belgium[2]; School of Pharmacy, University of Camerino, Italy[3]; VIB Center for Medical Biotechnology, Belgium[4]; Department of Biomolecular Medicine, Ghent University, Belgium[5]; VIB Proteomics Core, Belgium[6]; Department of Imaging and Pathology, KU Leuven, Belgium[7]; Department of Pathology, University Hospitals Leuven, KU Leuven, Belgium[8]; Translational Research Department, Institut Curie, PSL Research University, France[9]; Department of Obstetrics and Gynecology, University Hospitals Leuven, 3000 Leuven, Belgium[10]; Division of Oncogenomics, The Netherlands Cancer Institute, The Netherlands[11]; Centre for Gynecologic Oncology Amsterdam (CGOA), Antoni Van Leeuwenhoek-Netherlands Cancer Institute (AvL-NKI), University Medical Center (UMC), Amsterdam, The Netherlands[12]

* These authors equally contributed to this work

Supplementary information

(Figures and Methods)

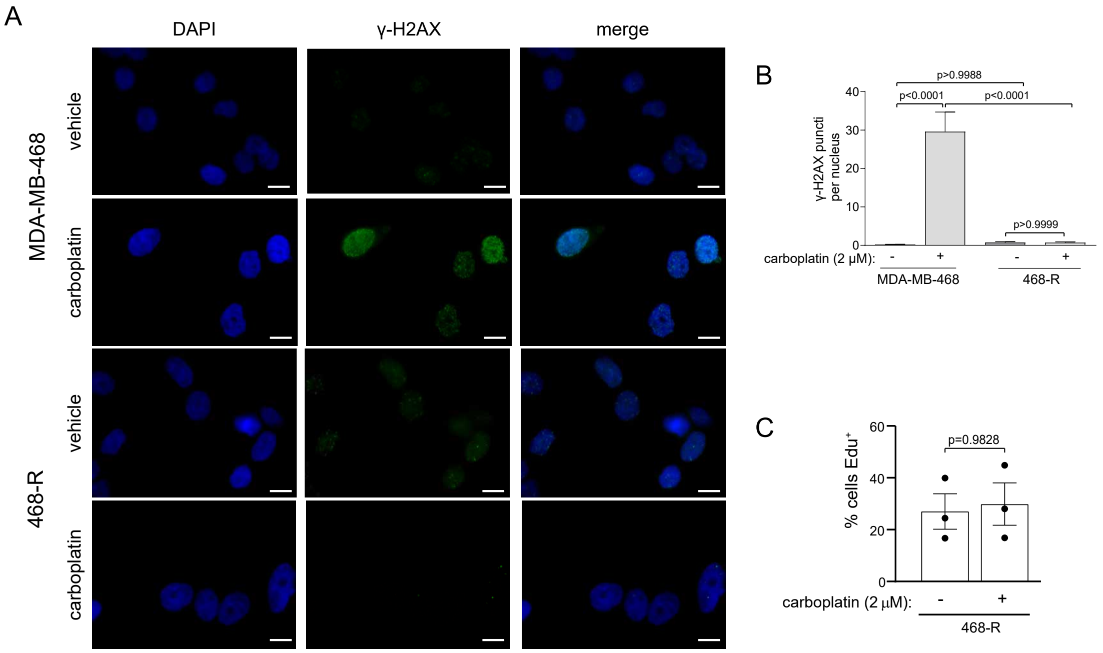

Figure S1. Carboplatin treatment of 468-R does not lead to increased DNA damage or affect DNA replication. A,B, γ-H2AX levels in MDA-MB-468 and 468-R cells treated with vehicle or 2 μM carboplatin for 5 days. Scale bar, 10 μm ***(A)*** and number of puncta per nucleus ***(B)***. N=3. Data are shown as Mean±SEM. P-values were calculated by two-way ANOVA with Geisser-Greenhouse and Tukey's corrections. **C,** EdU staining of 468-R cells. 468-R cells treated with vehicle or 2 μM carboplatin for 5 days were incubated with EdU for 2.5 hours. Data are shown as Mean±SEM. N=3. P-values were calculated by two-way ANOVA with Geisser-Greenhouse and Tukey's correction.

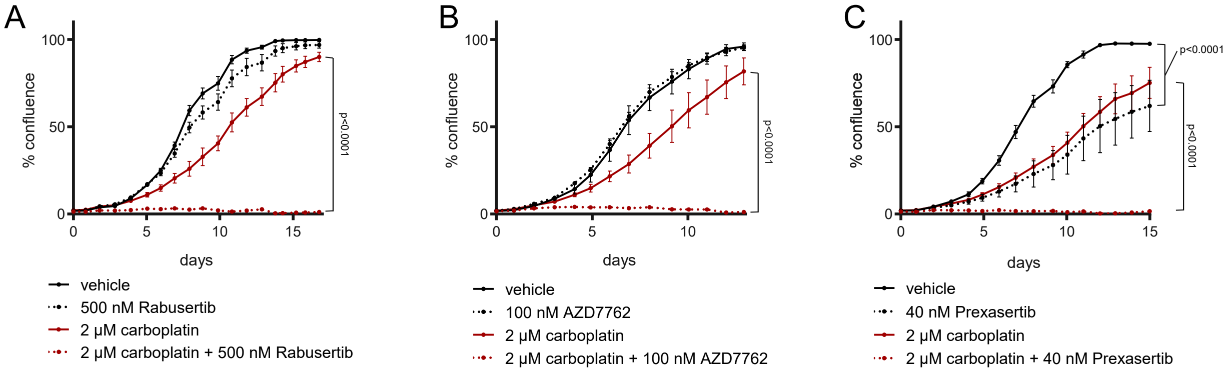

Figure S2. Inhibition of CHEK1 and WEE1 at high concentrations re-sensitize carboplatin-resistant 468-R cells, but have minimal effect as monotherapy. Carboplatin-resistant 468-R cells were treated with rabusertib (N=3) ***(A)***, AZD7762 (N=4) ***(B)***, and prexasertib (N=5) ***(C)*** for up to 17 days. Cell growth was analyzed as percent confluency by an Incucyte Imaging system. Data are shown as Mean±SEM. P-values versus carboplatin monotherapy or vehicle were calculated by two-way ANOVA with Geisser-Greenhouse and Dunnett's correction. Only significant P-values ($P < 0.05$) of the inhibitors are indicated.

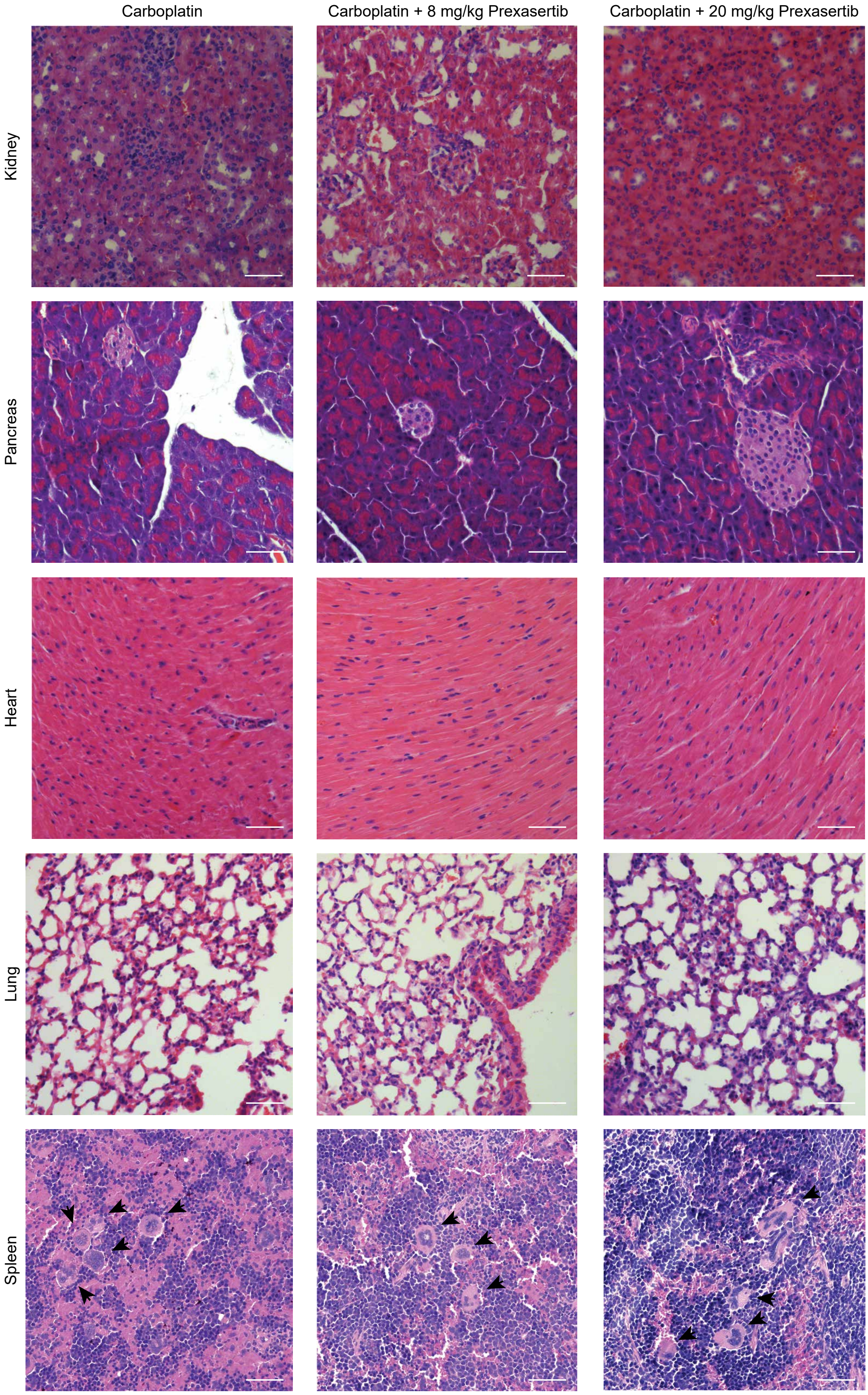

Figure S3. Neither low nor high dose Prexasertib affect the organs of healthy mice receiving carboplatin. NMRI nude mice were treated weekly with 50 mg/kg carboplatin plus either vehicle, low dose Prexasertib (8 mg/kg), or high dose Prexasertib (20 mg/kg). H&E stainings of kidney, pancreas, heart, lung, and spleen of mice treated with the indicated combination therapies. Notably no depletion of megakaryocytes was noticed in all the samples analyzed. Arrowheads indicate megakaryocytes. Scale bar = 50 µm. N=3

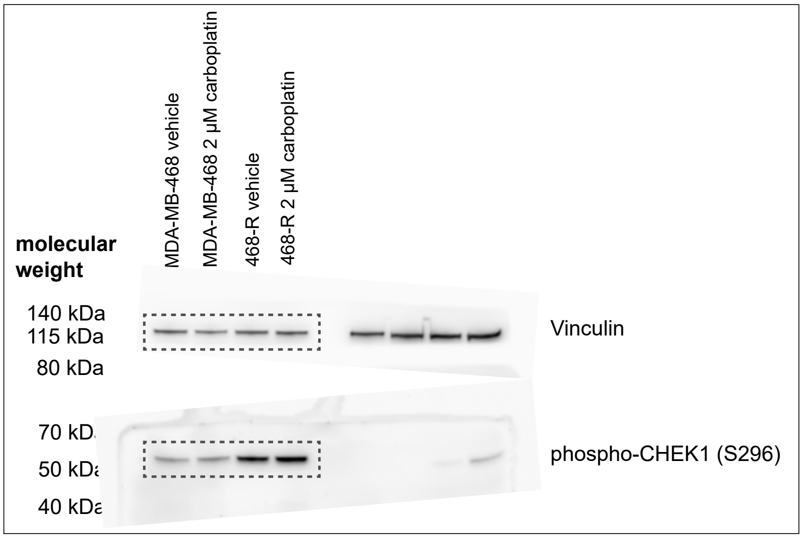

For Figure 3F

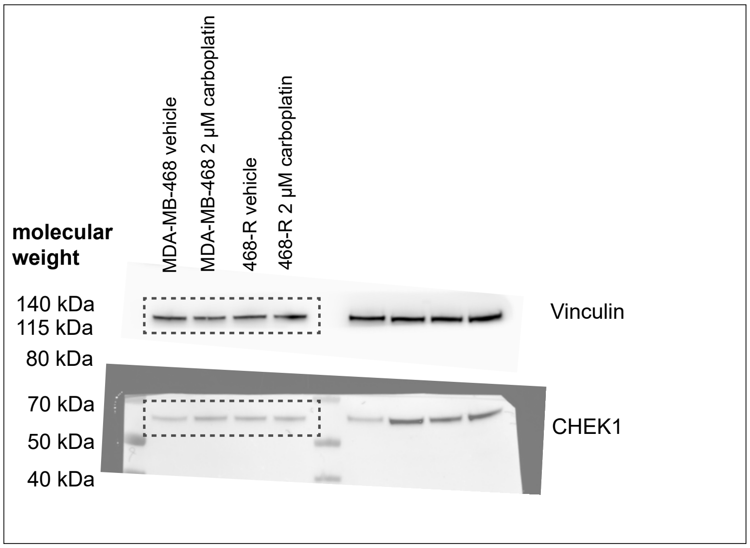

For Figure 3F

Figure S4. Full length blots used for Figure 3.

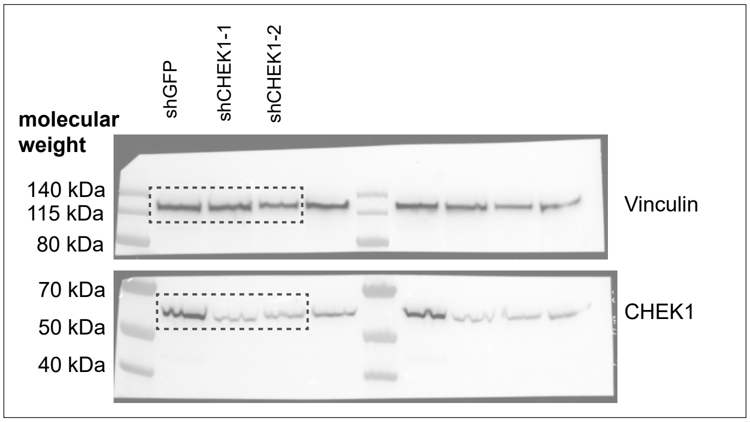

For Figure 4A

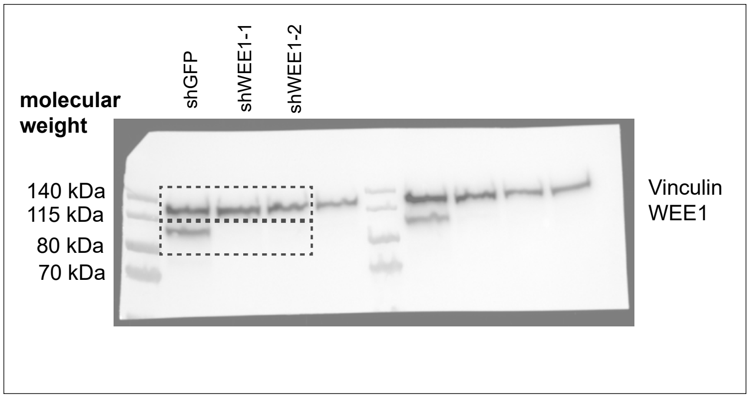

For Figure 4B

Figure S5. Full length blots used for Figure 4.

Supplemental Material and Methods

Liquid chromatography–tandem-mass spectrometry (LC-MS/MS)

Cell-pellets were dissolved in 1 ml lysis buffer (20 mM Hepes pH 8.0, 8 M urea) and sonicated with 3 pulses of 15 s at an amplitude of 20% using a 3 mm probe, with incubation on ice for 1 minute between pulses. After centrifugation for 15 minutes at 20,000 x g at room temperature to remove insoluble components, proteins were reduced by addition of 5 mM DTT and incubation for 30 minutes at 55˚C and then alkylated by addition of 10 mM iodoacetamide for 15 minutes at room temperature in the dark. The protein concentration was measured using a Bradford assay (Bio-rad) and from each sample 500 µg protein was used to continue the protocol. Samples were further diluted with 20 mM HEPES pH 8.0 to a final urea concentration of 4 M and proteins were digested with 5 µg LysC (Wako) (1/100, w/w) for 4 hours at 37°C. Samples were again diluted to 2 M urea and digested with 5 µg trypsin (Promega) (1/100, w/w) overnight at 37˚C. The resulting peptide mixture was acidified by addition of 1% trifluoroacetic acid (TFA) and after 15 minutes incubation on ice, samples were centrifuged for 15 minutes at 1,780 x g at room temperature to remove insoluble components. Next, peptides were purified on SampliQ SPE C18 cartridges (Agilent). Columns were first washed with 1 ml 100% acetonitrile (ACN) and pre-equilibrated with 3 ml of solvent A (0.1% TFA in water/ACN (98:2, v/v)) before samples were loaded on the column. After peptide binding, the column was washed again with 2 ml of solvent A and peptides were eluted twice with 750 µl elution buffer (0.1% TFA in water/ACN (40:60, v/v)).

Purified peptides were dried, re-dissolved in solvent A, peptide concentration was determined on a Lunatic spectrophotometer (Unchained Labs) and approximately 3 µg of each sample was injected for LC-MS/MS analysis on an Ultimate 3000 RSLCnano system (Thermo) in-line connected to a Q Exactive HF mass spectrometer (Thermo) equipped with a Nanospray Flex Ion source (Thermo). Trapping was performed at 10 µl/min for 4 min in solvent A on a 20 mm trapping column (made in-house, 100 µm internal diameter (I.D.), 5 µm beads, C18 Reprosil-

HD, Dr. Maisch, Germany) and the sample was loaded on a 400 mm analytical column (made in-house, 75 µm I.D., 1.9 µm beads C18 Reprosil-HD, Dr. Maisch). Peptides were eluted by a non-linear increase from 2 to 56% MS solvent B (0.1% formic acid (FA) in water/ACN (2:8, v/v)) over 140 minutes at a constant flow rate of 250 nl/min, followed by a 10-minutes wash reaching 99% MS solvent B and re-equilibration with MS solvent A (0.1% FA in water/ACN (2:8, v/v)). The column temperature was kept constant at 50°C (CoControl 3.3.05, Sonation). The mass spectrometer was operated in data-dependent, positive ionization mode, automatically switching between MS and MS/MS acquisition for the 16 most abundant peaks in a given MS spectrum. The source voltage was set to 2 kV and the capillary temperature was 250°C. Full-scan MS spectra (m/z 375-1500, AGC target 3E6 ions, maximum ion injection time of 60 ms), acquired at a resolution of 60,000 (at 200 m/z) was followed by up to 16 tandem MS scans, acquired at a resolution of 15,000 (at 200 m/z) of the most intense ions fulfilling predefined selection criteria: AGC target 1E5 ions, maximum ion injection time of 80 ms, isolation window of 1.5 m/z, fixed first mass of 145 m/z, spectrum data type: centroid; under fill ratio 2%; intensity threshold 1.3E4; exclusion of unassigned and singly charged precursors; peptide match preferred; exclude isotopes on; dynamic exclusion time of 12 s. The normalized collision energy was set to 28% and the polydimethylcyclosiloxane background ion at 445.12003 Da was used for internal calibration (lock mass). QCloud was used to control instrument longitudinal performance during the project.

Data analysis was performed with MaxQuant (version 1.6.1.0) using the Andromeda search engine with default search settings including a false discovery rate set at 1% on both the peptide and protein level. Spectra were searched against the human proteins in the Swiss-Prot database (database release version of January 2018 containing 20,231 human protein sequences, www.uniprot.org). The mass tolerance for precursor and fragment ions was set to 4.5 and 20 ppm, respectively, during the main search. Enzyme specificity was set as C-terminal to arginine and lysine, also allowing cleavage at proline bonds with a maximum of two missed cleavages. Variable modifications were set to oxidation of methionine residues and acetylation

of protein N-termini, while carbamidomethylation of cysteine residues was set as fixed modification. Matching between runs was enabled with a matching time window of 1.5 minutes and an alignment time window of 20 minutes. Only proteins with at least one unique or razor peptide were retained leading to the identification of 5,541 proteins. Proteins were quantified by the MaxLFQ algorithm integrated in the MaxQuant software. A minimum ratio count of two unique or razor peptides was required for quantification.

Further data analysis was performed with the R software after loading the proteingroups file from MaxQuant. Reverse database hits were removed and replicate samples were grouped. Proteins that presented in all replicates of at least one condition were kept. LFQ intensity values were log2 transformed and k-Nearest Neighbour was used to impute missing values by R package 'VIM'. To identify the dysregulated proteins between conditions, ANOVA followed by Tukey's HSD Test was performed. Significantly differentially expressed proteins were determined by adjusted p-value < 0.05. Proteins that were significantly differentially expressed were selected for the clustering analysis using the ‘Euclidean’ distance measure and ‘ward.D2’ clustering method.
